# Characterization of adipocyte differentiation from human mesenchymal stem cells in bone marrow

**DOI:** 10.1186/1471-213X-10-47

**Published:** 2010-05-07

**Authors:** Shu-Wen Qian, Xi Li, You-You Zhang, Hai-Yan Huang, Yuan Liu, Xia Sun, Qi-Qun Tang

**Affiliations:** 1 Institute of Stem Cell Research and Regenerative Medicine, Institutes of Biomedical Sciences, Fudan University, Shanghai 200032, PR China; 2 The Key Laboratory of Molecular Medicine, the Ministry of Education, Department of Biochemistry and Molecular Biology, Shanghai Medical College, Fudan University, Shanghai 200032, PR China

## Abstract

**Background:**

Adipocyte hyperplasia is associated with obesity and arises due to adipogenic differentiation of resident multipotent stem cells in the vascular stroma of adipose tissue and remote stem cells of other organs. The mechanistic characterization of adipocyte differentiation has been researched in murine pre-adipocyte models (i.e. 3T3-L1 and 3T3-F442A), revealing that growth-arrest pre-adipocytes undergo mitotic clonal expansion and that regulation of the differentiation process relies on the sequential expression of three key transcription factors (C/EBPβ, C/EBPα and PPARγ). However, the mechanisms underlying adipocyte differentiation from multipotent stem cells, particularly human mesenchymal stem cells (hBMSCs), remain poorly understood. This study investigated cell cycle regulation and the roles of C/EBPβ, C/EBPα and PPARγ during adipocyte differentiation from hBMSCs.

**Results:**

Utilising a BrdU incorporation assay and manual cell counting it was demonstrated that induction of adipocyte differentiation in culture resulted in 3T3-L1 pre-adipocytes but not hBMSCs undergoing mitotic clonal expansion. Knock-down and over-expression assays revealed that C/EBPβ, C/EBPα and PPARγ were required for adipocyte differentiation from hBMSCs. C/EBPβ and C/EBPα individually induced adipocyte differentiation in the presence of inducers; PPARγ alone initiated adipocyte differentiation but the cells failed to differentiate fully. Therefore, the roles of these transcription factors during human adipocyte differentiation are different from their respective roles in mouse.

**Conclusions:**

The characteristics of hBMSCs during adipogenic differentiation are different from those of murine cells. These findings could be important in elucidating the mechanisms underlying human obesity further.

## Background

Increased adipose tissue mass associated with obesity is due to the increased number and size of adipocytes [1,2]. Adipocyte differentiation from mesenchymal stem cells plays an important role in the hyperplasia of adult adipose tissue. A population of cells resident in the vascular stroma of adipose tissue can differentiate into adipocytes *in vitro *and *in vivo *[3]. Recent studies indicate that pericytes in blood vessel walls have adipogenic potential, express mesenchymal stem cell (MSC) markers and are multipotent [4]. In addition to resident stem cells, non-resident stem cells can serve as a source of adipocyte precursors; bone marrow MSCs can be recruited to adipose tissue and generate new adipocytes in response to treatment with thiazolidinediones (TZDs) or high fat stimulation [5].

The characteristics and molecular mechanism underlying adipocyte differentiation have been extensively investigated in the murine pre-adipocyte cell lines 3T3-L1 and 3T3-F442A [6,7]. Growth-arrested pre-adipocytes have been shown to re-enter the cell cycle synchronously and undergo mitotic clonal expansion in response to MDI (M: methyl-isobutyl-xanthine, D: dexamethasone, I: insulin) treatment, before exiting the cell cycle and terminally differentiating [8]. The transcription factors C/EBPβ (CCAAT/enhancer binding protein β), C/EBPα (CCAAT/enhancer binding protein α) and PPARγ (peroxisome proliferator-activated receptor γ) act sequentially during 3T3-L1 pre-adipocyte differentiation [9]. C/EBPβ is induced immediately after exposure to the differentiation cocktail, resulting in phosphorylation and activation [10,11], and it transactivates the expression of C/EBPα and PPARγ [12]. C/EBPα and PPARγ, together or in isolation, can initiate differentiation without inducers [13-15]. C/EPBα is believed to be relevant to the acquisition of insulin sensitivity [16].

MSCs have been isolated and induced to differentiate into adipocytes in a variety of organs [17-22]. However, the differentiation procedure and the roles of adipose-related genes in that procedure have not been characterized completely owing to the heterogeneity, low proliferation ability and ineffective ectopic gene transfection of hBMSCs [23,24]. Human primary cells are of great interest because of their biological and therapeutic potential, therefore this study extends the research carried out in murine 3T3-L1 cells to hBMSCs from bone marrow.

## Results

### Isolation and adipogenic differentiation of hBMSCs

**Figure 1:**
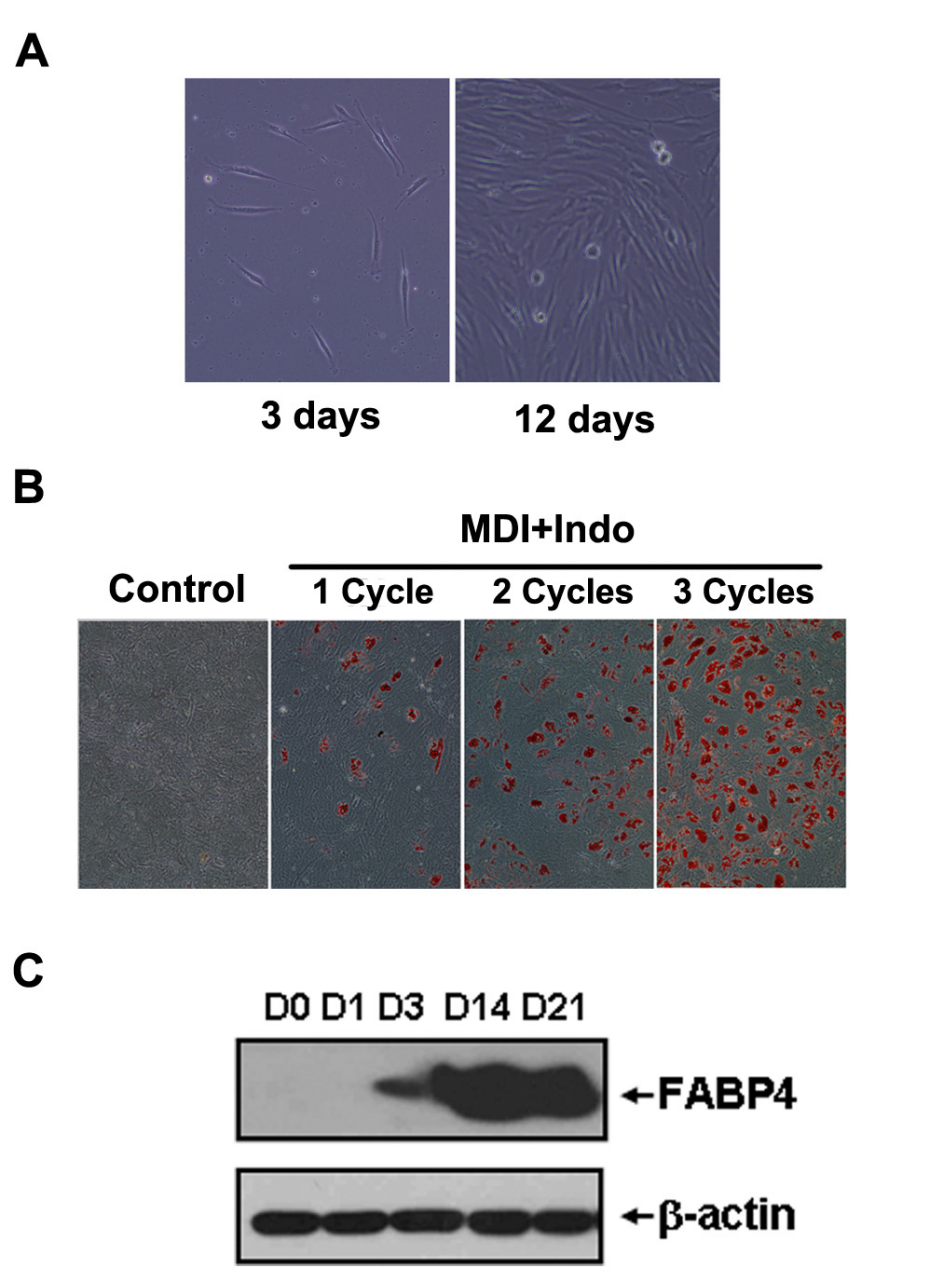
**Isolation and adipogenic differentiation of hBMSCs**. (A) The morphology of adherent hBMSCs three and 12 days after plating (magnification 100×). (B) HBMSCs of P5 were cultured for one week after confluence and induced to differentiate with MDI+Indo (M: methyl-isobutyl-xanthine; D: dexamethasone; I: insulin; Indo: indomethacin) treatment for one, two or three cycles (1 cycle of treatment: MDI+Indo for three days followed by insulin for one day). The accumulation of cytoplasmic triglyceride was detected by Oil Red O staining on day 21 and visualized under a microscope (magnification 100×). (C) FABP4 expression was examined by Western Blotting at the indicated days after differentiation with repeated MDI+Indo treatment (three times).

Isolated hBMSCs presented with a typical spindle-shape phenotype (Figure 1A), and cells from passages 3-5 were used for the following studies. In addition to fetal bovine serum (FBS), methyl-isobutyl-xanthine, dexamethasone and insulin (MDI) used to induce 3T3-L1 adipocyte differentiation, indomethacin (Indo), a PPARγ agonist [25], was added to the culture medium (MDI+Indo) to induce adipocyte differentiation from hBMSCs [26]. Each cycle of MDI+Indo threatment only induced a portion of hBMSCs to go into adipocyte differentiation, and about 60%-70% hBMSCs differentiated into adipocytes after three cycles of MDI+Indo induction as indicated by oil red O staining (Figure 1B). Consistent with the morphological changes, the expression of the adipose-specific gene FABP4 (422/aP2 in mouse) was significantly induced throughout differentiation as determined by Western Blotting (Figure 1C).

### Cell cycle alteration during adipocyte differentiation from hBMSCs

**Figure 2:**
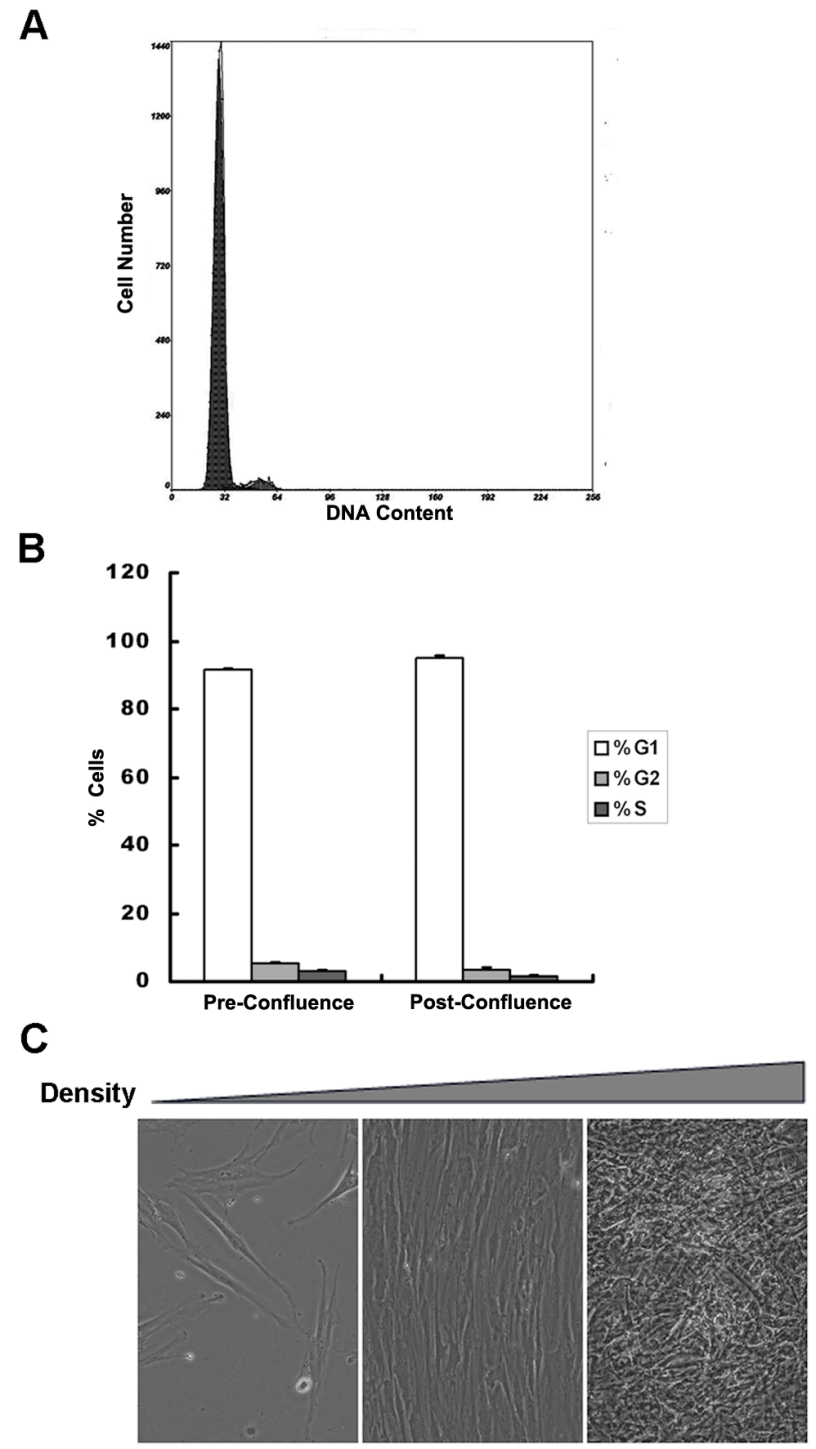
**Growth characteristic of hBMSCs**. (A) Confluent hBMSCs were trypsinized, fixed and stained with PI. DNA content in cells was examined by flow cytometry. (B) Pre-confluent (density ~80%) and post-confluent (one week after cells reach confluence) hBMSCs from three separate experiments at different cell cycle stages revealed by flow cytometry were quantified. (C) Morphology of hBMSCs at different densities (plated at 5000/cm^2 ^and cultured for one day, one week and five weeks).

HBMSCs proliferated slowly, approximately <10% of cells were actively dividing revealed by DNA content with flow cytometry (Fig.2A). About 90% of the cells in G0/G1 phase were at the dividing stage, and approximately 95% at the post-confluence stage (Figure 2B). Contact inhibition was not apparent, as observed that if plated at a density of 5000 cells/cm^2 ^and cultured for five weeks, the cells locally grew into multi-layers (Figure 2C).

**Figure 3:**
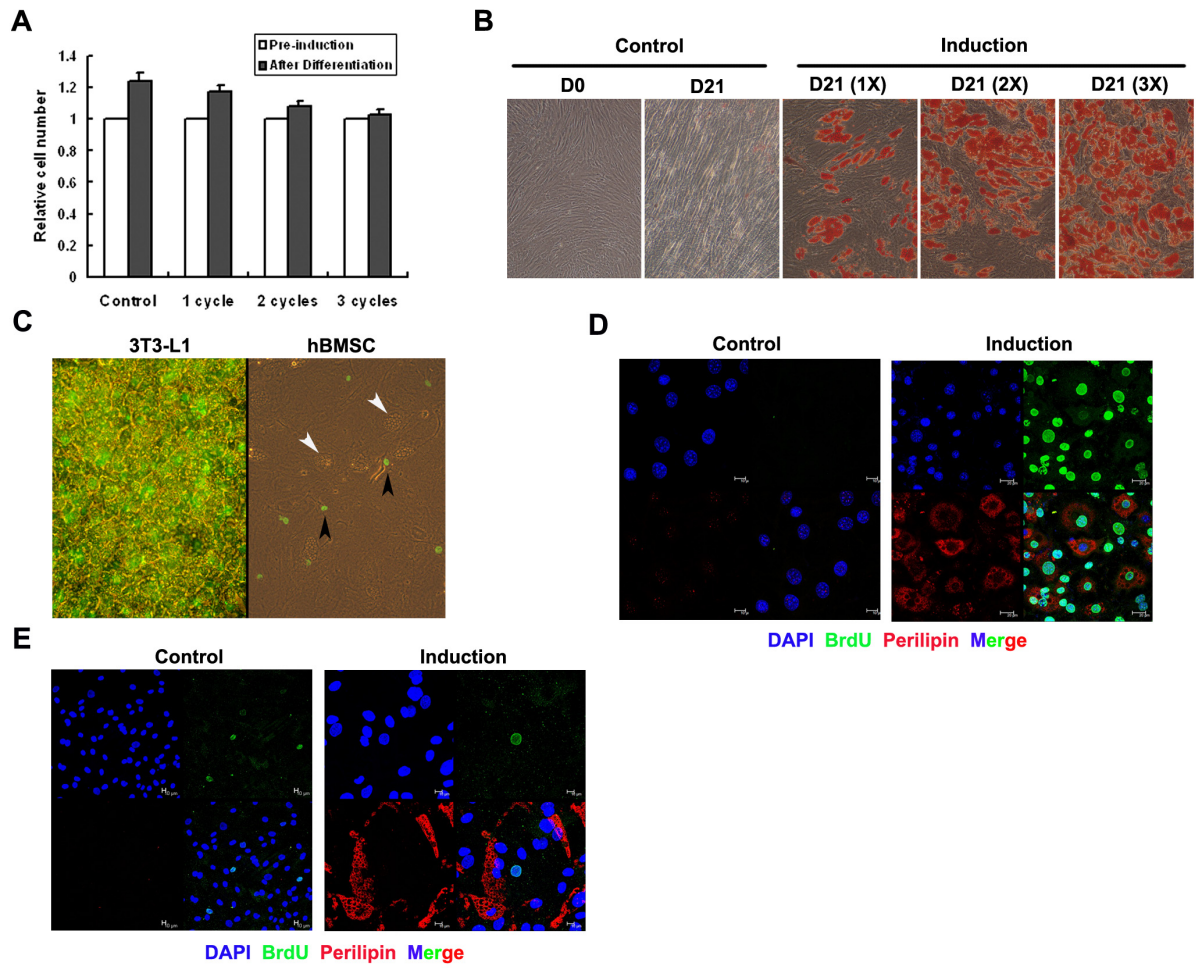
**Cell cycle progression during adipogenic differentiation of hBMSCs**. (A) Post-confluence hBMSCs with or without induction (MDI+Indo treatment for one, two or three cycles) were counted and plotted on day 0 and day 21. (B) Cells with parallel treatment in (A) were also stained with oil red O on day 21 and photographed (magnification 100×). (C) 10 μg/ml BrdU was added to 3T3-L1 cells at 18 h after MDI treatment for 30 h, and added to hBMSC at 24 h for 48 h. BrdU incorporation was detected by immunocytochemistry and photographed with both a halogen and mercury lamp switched on (magnification 200×). In 3T3-L1 cells (D) and hBMSCs (E) with or without induction (control), incorporated BrdU (FITC) and fat lipids (TRITC) were shown by confocal microscopy.

Cell cycle regulation is an important event in adipocyte differentiation of mouse 3T3-L1 pre-adipocytes [8,11]. Growth-arrested 3T3-L1 pre-adipocytes synchronously re-enter the cell cycle upon MDI induction and undergo two rounds of division before expression of adipocyte-specific genes and presenting with the mature adipocyte phenotype. In order to investigate whether hBMSCs undergo division during adipocyte differentiation, the number of cells was counted (Figure 3B). The cell number marginally increased (1.24 fold) in the control group after a 21-day culture (Figure 3A). There was an increase in the cell number (1.17 fold) in differentiation cultures but less than that in the control, and as the number of differentiated cells increased after repeated inductions, the rate of increase of cell numbers declined. These results suggest that the proliferation of undifferentiated cells contributed to the increase in cell numbers.

BrdU incorporation assays were performed to investigate whether DNA synthesis occurs during adipocyte differentiation from hBMSCs. We found that differentiated hBMSCs were BrdU negative, while differentiated 3T3-L1 cells were BrdU positive (Figure 3C). Confocal microscopy verified the positional relationship between nuclei (as indicated by BrdU incorporation into DNA) and cells with lipid droplets in the cytoplasm (Figure 3D, 3E). These results demonstrate that hBMSCs did not undergo mitotic clonal expansion during adipogenic differentiation under culture conditions.

### Role of C/EBPβ in adipocyte differentiation of hBMSCs

**Figure 4:**
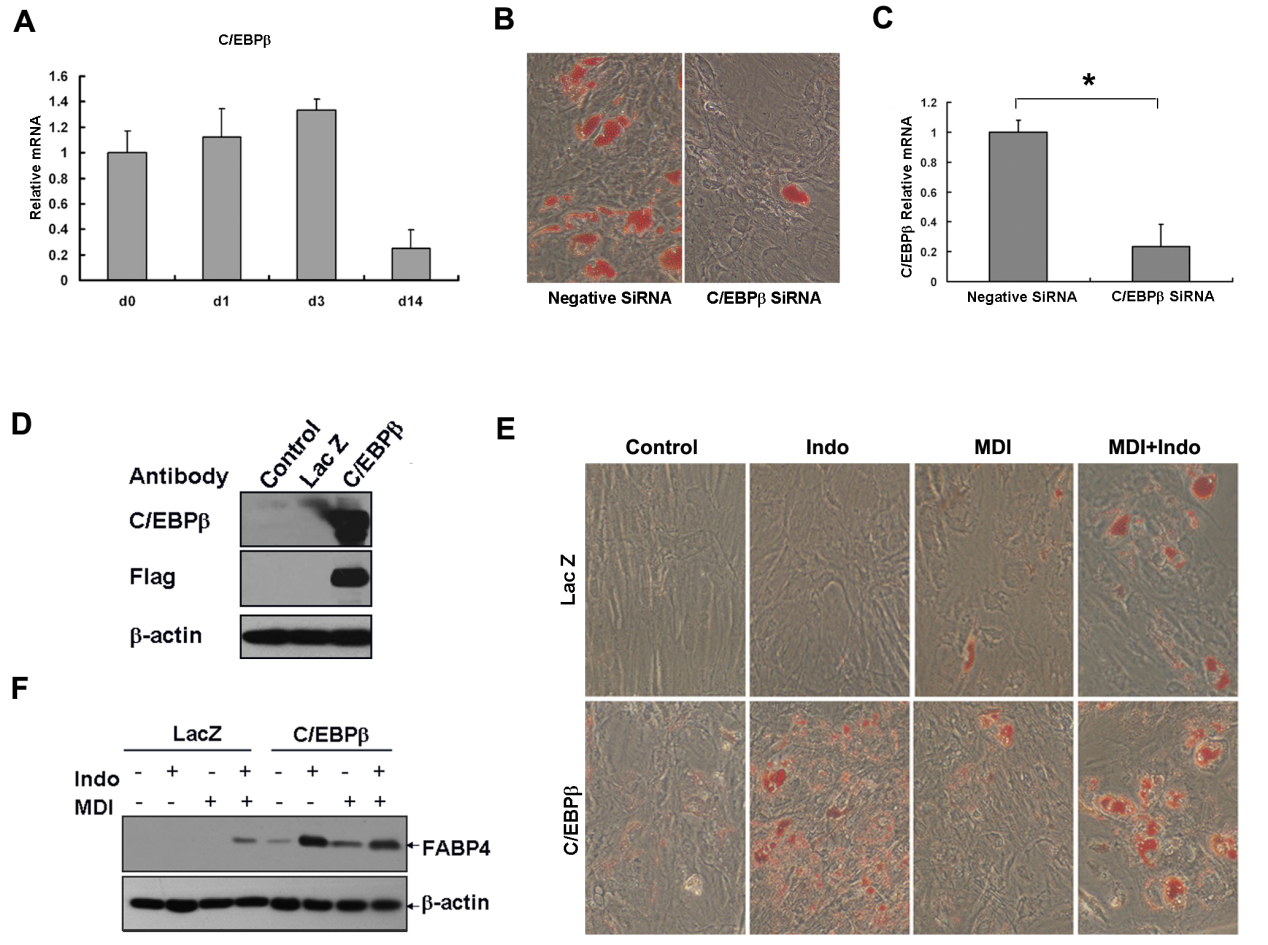
**C/EBPβ was required for and stimulated adipocyte differentiation from hBMSCs**. (A) Relative expression levels of C/EBPβ were determined at the indicated days by real-time PCR. (B) Adipogeic differentiation revealed by oil red O staining with C/EBPβ knock-down by SiRNA. (C) Expression levels of C/EBPβ were determined by real-time PCR (n = 3, *P < 0.05). (D) Over-expression of C/EBPβ in hBMSCs with adenoviral infection (Lac Z as control) was confirmed by Western Blotting. (E) HBMSCs were cultured to confluence and infected with adenovirus at MOI 10 followed by various combination of hormone treatment 4 h later for three days. Cells were stained with oil red O on day eight (magnification 100×). (F) The expression of the adipocyte marker (FABP4) was detected on day four by Western Blotting.

In order to define the role of C/EBPβ in adipocyte differentiation of hBMSCs, the expression profile was determined. Expression of C/EBPβ in hBMSCs could be detected at the start of induction by real-time PCR; the expression level did not change significantly during the early stages of induction (Figure 4A) but declined after 14 days when most of the cells had differentiated. Regarding the expression difference between 3T3-L1 and hBMSCs, C/EBPβ expression was knocked down by siRNA to determine whether C/EBPβ is essential during adipocyte differentiation from hBMSCs; and knocked-down expression of C/EBPβ was confirmed by real-time PCR (Figure 4C). HBMSCs failed to differentiate into adipocytes after C/EBPβ was knocked down (Figure 4B).

C/EBPβ was over-expressed in hBMSCs using an adenovirus expression system (Figure 4D) to investigate its function during differentiation. Control cells expressing Lac Z didn't differentiate, while expression of exogenous C/EBPβ alone induced adipogenesis (Figure 4E), and some cells presented with small intracellular fat droplets that could not be adequately stained using oil red O. However, FABP4 expression was detected by western blotting (Figure 4F) and was significantly up-regulated by the addition of inducers, the highest levels of expression being evident when indomethacin (PPARγ agonist) was included (Figure 4E, 4F).

### Role of C/EBPα in adipocyte differentiation from hBMSCs

**Figure 5:**
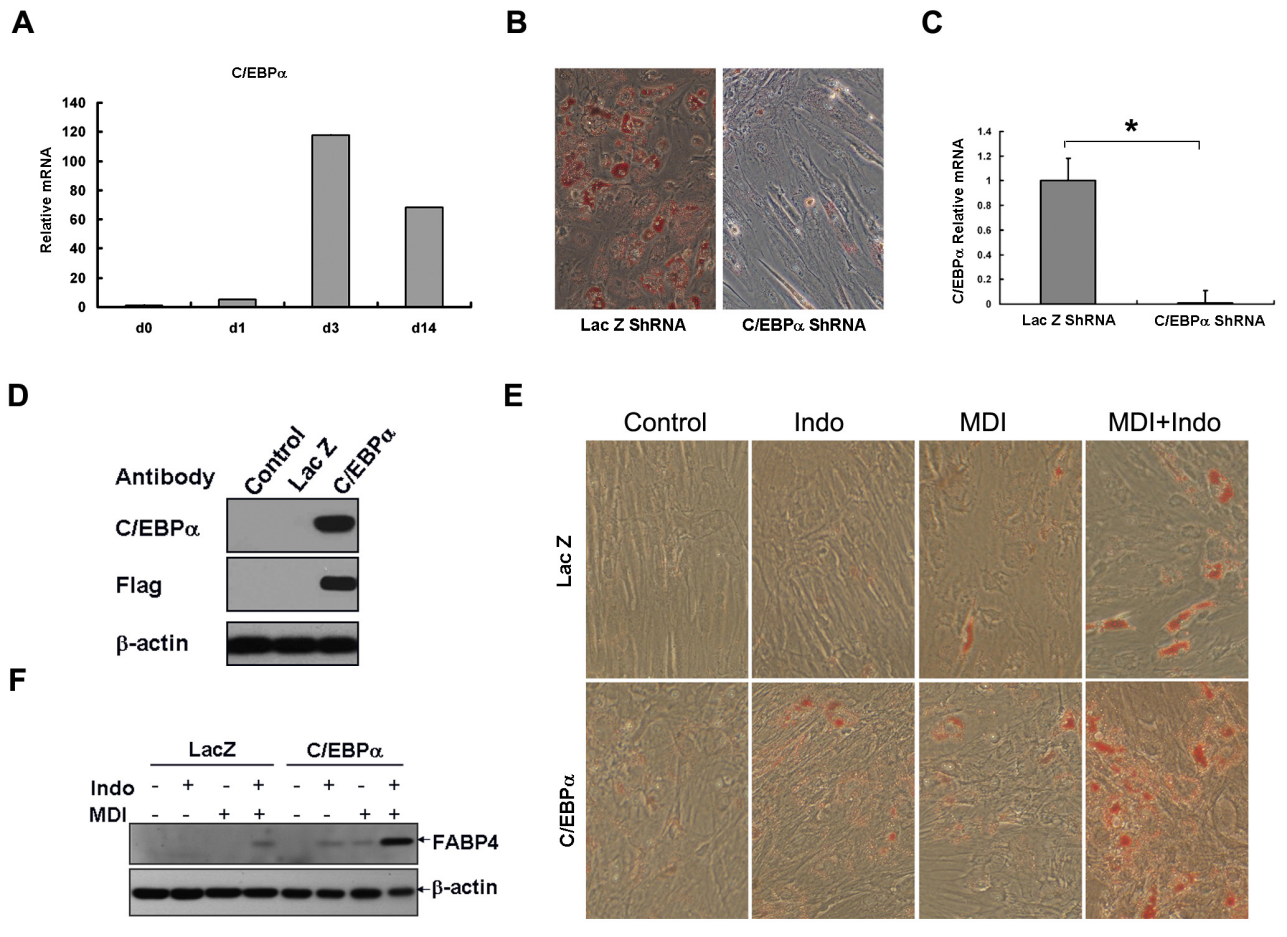
**C/EBPα was required for and stimulated adipocyte differentiation from hBMSCs**. (A) Relative expression levels of C/EBPα were determined at the indicated days by real-time PCR. (B) Adipocyte differentiation revealed by oil red O staining with C/EBPα knock-down by adenovirus expressing shRNA. (C) C/EBPα knock-down was confirmed by real-time PCR (n = 3, *P < 0.05). (D) C/EBPα over-expression in hBMSCs using adenovirus (Lac Z as control) was shown by Western Blotting. (E) HBMSCs were cultured to confluence and infected with adenovirus at MOI 10 followed by various combinations of hormone treatment 4 h later for three days. Cells were stained with oil red O on day eight (magnification 100×). (F) The expression of the adipocyte marker (FABP4) was detected on day four by Western Blotting.

Expression of C/EBPα increased one day after induction, reached a maximum level after three days and decreased by day 14 when adipocyte differentiation had occurred (Figure 5A). As Figure 5B demonstrates, knocked-down expression of C/EBPα (Figure 5C) with an adenovirus carrying C/EBPα shRNA impaired the differentiation of hBMSCs, while over-expression of C/EBPα (Figure 5D) in hBMSCs did not induce adipocyte differentiation. However, C/EBPα together with indomethacin or MDI induced a small proportion of the cells to differentiate, and when both indomethatin and MDI were added with C/EBPα, the number of differentiated adipocytes increased as demonstrated by oil red O staining (Figure 5E) and Western Blotting of FABP4 expression (Figure 5F).

### Role of PPARγ in adipocyte differentiation from hBMSCs

**Figure 6:**
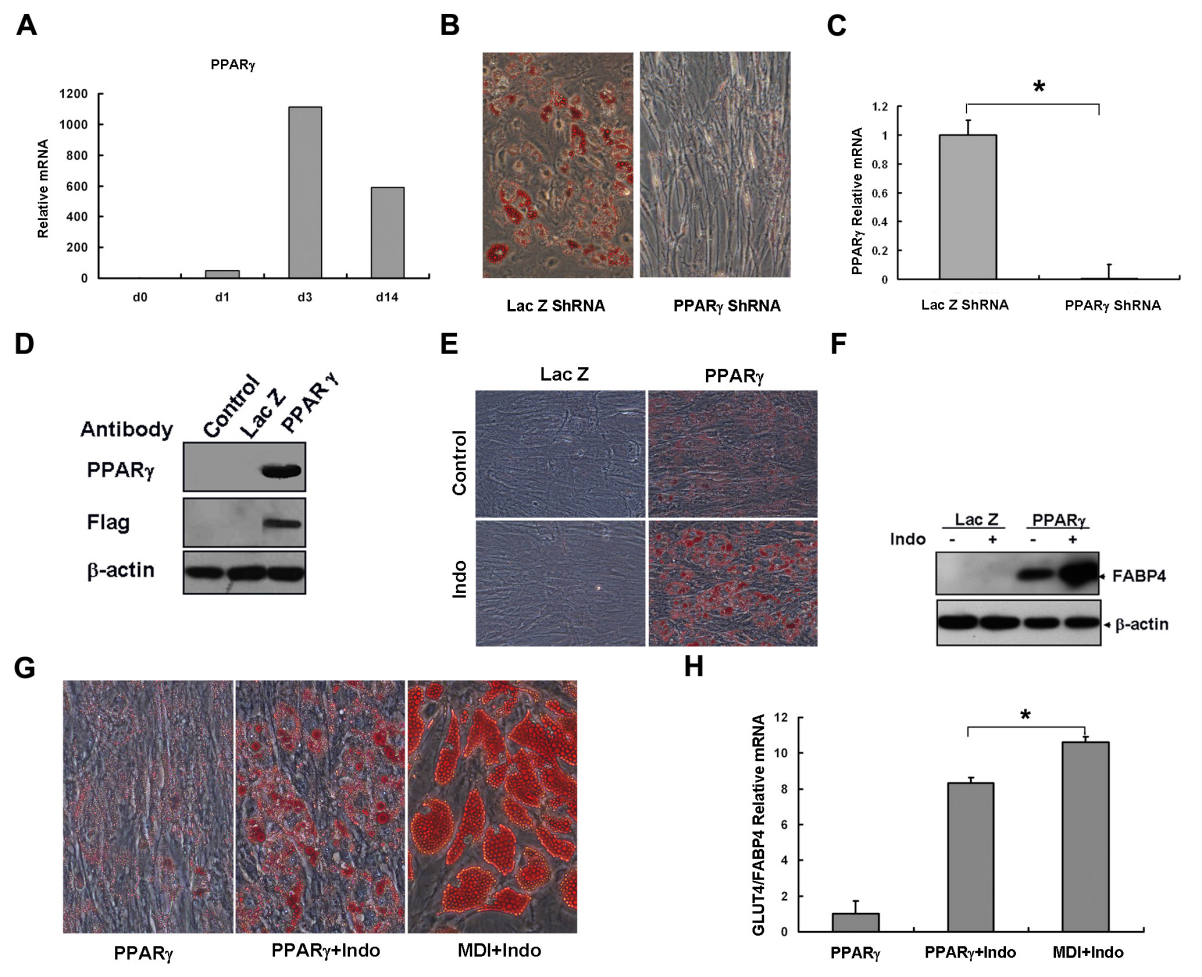
**PPARγ was sufficient to initiate adipocyte differentiation from hBMSCs, but could not induce fully developed adipocytes**. (A) Relative expression levels of PPARγ were determined at the indicated days by real-time PCR. (B) Adipogeic differentiation revealed by oil red O staining with PPARγ knock-down by adenovirus expressing shRNA. (C) PPARγ knock-down was verified by real-time PCR (n = 3, *P < 0.05). (D) PPARγ over-expression in hMBSCs with adenovirus (Lac Z as control) was shown by Western Blotting. (E) HBMSCs were cultured to confluence and infected with adenovirus at MOI 10 alone or in combination with indomethacin. Lipid droplets indicated by oil red O staining on day 14 (magnification 100×). (F) The expression of adipocyte marker FABP4 was detected on day six by Western Blotting. (G) Morphology of lipid droplets induced by PPARγ expression and hormone treatment (magnification 200×). (H) GLUT4 expression normalized by FABP4 was quantified by real-time PCR in cells treated with PPARγ adenovirus or hormone (n = 3, *P < 0.05).

The induction of PPARγ expression was similar to that of C/EBPα (Figure 6A, Figure 5A). Knock-down of PPARγ expression in hBMSCs (Figure 6C) prevented adipocyte differentiation (Figure 6B), while over-expression of PPARγ (Figure 6D) induced adipogenic differentiation (Figure 6E), resulting in fat droplet accumulation in the vast majority of cells. Addition of an exogenous PPARγ agonist (indomethacin) enhanced the function of PPARγ as determined by oil red O staining (Figure 6E) and FABP4 expression (Figure 6F). Fat droplets appeared three days after adenoviral infection but were smaller than those induced by MDI+Indo (Figure 6G). The expression ratio of GLUT4 to FABP4 in adipocytes induced by PPARγ over-expression was lower than that in cells induced by three cycles of MDI+Indo (Figure 6H).

## Discussion

HBMSCs are more difficult to handle than mouse stem cell lines but their importance and therapeutic potential necessitate their use in research of the type outlined herein. The previous studies are focused on the mouse stem cell lines but the regulation of them could be different in some aspects, and results of murine cells would be less convincing in interpreting the onset of human disease. On the other hand, adipocytes differentiated from HBMSCs would be of better immuno-compatibility in autograft for plastic purpose. So, in this study, a comprehensive analysis of adipocyte differentiation from multipotent human stem cells was carried out.

HBMSCs were isolated from bone marrow and induced to differentiate into adipocytes under culture conditions. The PPARγ agonist, indomethacin, was added as well as the conventional inducers used in adipocyte differentiation protocols for murine pre-adipocytes. HBMSCs behaved differently from 3T3-L1 pre-adipocytes, with only a small number of cells differentiating into adipocytes after one cycle of treatment; approximately 60%~70% of hBMSCs differentiated into adipocytes after three cycles of treatment (Figure 1B). A long G0 phase and a lack of contact inhibition (Figure 2C) meant that hBMSCs did not synchronize at the time when differentiation was initiated (Figure 2B). Growth arrest is a prerequisite for adipocyte differentiation [27], so it was concluded that only a minority of hBMSCs were growth arrested when differentiation was induced.

MCE (mitotic clonal expansion) is an essential event associated with adipocyte differentiation from mouse pre-adipocyte cell lines [8,11]. However, it is not known whether MCE is required for adipocyte differentiation from all cell types. We have previously demonstrated that committed C3H10T1/2 cells treated with BMP4 divide when induced to differentiate [28], and primary cultures of mouse embryonic fibroblasts (MEF) undergo MCE when differentiating into adipocytes [29]. In this study, hBMSCs from bone marrow did not undergo division during differentiation (Figure 3), which is in agreement with other reports showing that adipose precursor cells prepared from human adipose tissue (hADSCs) did not divide during differentiation under culture conditions [30]. The authors argued that hADSCs had completed division before being isolated; however, hADSCs are multipotent and can differentiate into other cell lineages including adipocytes ex vivo [31,32]. HADSCs could behave similarly to hBMSCs from bone marrow under culture conditions and remain uncommitted. The diversity of cell cycle alterations during adipocyte differentiation could be species-specific.

Murine proteins and comparable human proteins can function differently in the same context. In this study, C/EBPβ expression in hBMSCs did not alter significantly during the early stages of induction whereas expression was up-regulated immediately following induction and declined after two days in 3T3-L1 pre-adipocytes [33]. The decline of C/EBPβ at 14 day might result from most of cells being terminal differentiated. However, C/EBPβ was required for adipocyte differentiation in hBMSCs as its knock-down expression impaired differentiation (Figure 4B, 4C). C/EBPβ has important roles in mitosis and terminal adipocyte differentiation [34,35], but mitosis did not occur during differentiation of hBMSCs (Figure 3) and that could possibly explain the lack of differential expression of C/EBPβ upon induction. It is likely that the role of C/EBPβ during adipocyte differentiation from hBMSCs relates to its modification and not its expression levels, although the importance of C/EBPβ phosphorylation requires further investigation.

C/EBPβ or C/EBPα is sufficient to induce 3T3-L1 pre-adipocytes to differentiate into mature adipocytes without using inducers [36,37]. Over-expression of C/EBPβ alone stimulated differentiation of hBMSCs, as evidenced by FABP4 expression (Figure 4E, 4F). C/EBPα was less effective than C/EBPβ as expression of C/EBPα alone did not stimulate differentiation (Figure 5E, 5F). C/EBPβ and C/EBPα individually enhanced adipocyte differentiation of hBMSCs dependent on exogenous hormone agent treatment, particularly in the presence of a PPARγ activator (Figure 4E, 4F, 5E, 5F). HBMSCs may lack endogenous PPARγ ligands; however, this cannot be determined at this time because the results concerning the study of natural PPARγ ligands are indecisive [38].

PPARγ plays pivotal roles in adipocyte differentiation as it induces adipogenesis in cultured mouse fibroblasts [14]. With the use of high affinity, selective PPARγ agonists, PPARγ activation stimulates 3T3-F442A cells to develop into mature fat cells with a phenotype that includes morphological changes, lipid accumulation, and the acquisition of insulin sensitivity [39]. In addition, ectopic expression of PPARγ in hBMSCs initiates adipocyte differentiation. However, these cells were immature adipocytes, as demonstrated by morphological observations and the expression of some adipocyte-specific genes (Figure 6G, 6H). In humans, PPARγ functions to regulate a part of genes required for adipocyte maturation, as demonstrated by its ability to induce FABP4 but not GLUT4 expression (Figure 6H). In addition, PPARγ could play a role in cytoskeletal alterations associated with the morphological changes during differentiation, as the cells rounded up when PPARγ was over-expressed and elongated when expression of PPARγ was knocked down.

## Conclusions

This study demonstrates that the characteristics of hBMSCs during adipogenic differentiation are different from those of mouse cells. HBMSCs do not undergo mitotic clonal expansion during adipocyte differentiation. C/EBPβ, C/EBPα, and PPARγ are all required but not sufficient for adipocyte differentiation from hBMSCs. The ability of the transcription factors to stimulate adipocyte differentiation differed between human and murine cells. Further studies concerning on how C/EBPβ, C/EBPα and PPARγ regulating human adipocyte differentiation could help to elucidate the molecular mechanism of adipocyte differentiation from human stem cells, help to elucidate the mechanisms underlying human obesity and identify therapeutic targets.

## Methods

### Donor information

Bone marrow was obtained from the iliums of patients undergoing iliac crest bone grafts following informed consent. Five samples were obtained from male patients between the ages of 25 and 55 years who did not suffer from obesity and/or diabetes. The sample collection procedure and related research work was approved by the ethics committee of Institutes of Biomedical Sciences, Fudan University. Results were reproducible between donors, and the data presented in the results section were from a 32-year-old male donor.

### Isolation and adipogenic differentiation of hBMSCs

HBMSCs were isolated by density gradient centrifugation with Ficoll-Paque (GE Healthcare) and plastic adherence and grown in DMEM (low glucose, Invitrogen) containing 10% fetal bovine serum and 1% antibiotics; cells from passages 3-5 were used experimentally. A published protocol was followed to induce adipogenic differentiation of hBMSCs [26]. HBMSCs were cultured at a density of 5000~6000 cells/cm^2^. After reaching confluence, hBMSCs were cultured for one more week and induced in adipogenic medium containing 0.5 mM isobutyl-methylxanthine (Sigma-Aldrich), 1 μM dexamethasone (Sigma-Aldrich), 10 μM insulin (Roche), 100 μM indomethacin (Sigma-Aldrich) for three days and maintained in medium with 10 μM insulin for one day. The treatment was repeated two or three times, after which the cells were maintained in DMEM with 10 μM insulin until day 21 and subjected to oil red O staining to detect cytoplasmic triglyceride.

### Oil red O staining

Cells were washed three times with PBS and then fixed for 2 min with 3.7% formaldehyde. Oil red O (0.5% in isopropanol) was diluted with water (3:2) filtered through a 0.45 μm filter and incubated with the fixed cells for 1 h at room temperature. Cells were washed with water and the stained fat droplets in the cells were visualized by light microscopy and photographed. The percentage of differentiated cells was determined by counting cells based on oil red staining in the lipid vacuoles and 4',6'-diamidino-2-phenylindole staining of DNA.

### Western blotting

At various time points cells were washed with cold PBS (pH 7.4) and lysed with lysis buffer (2% SDS, 60 mM Tris-Cl, pH 6.8). The lysates were heated to 100°C for 10 min and clarified by centrifugation; equal amounts of protein were separated by SDS-PAGE. Proteins were transferred to poly(vinylidene difluoride) membranes and immunoblotted with antibodies to FABP4(422/aP2), C/EBPβ, C/EBPα, and PPARγ [antibodies to 422/aP2, C/EBPβ and C/EBPα were provided by Dr. M Daniel Lane (Johns Hopkins University School of Medicine, Baltimore) and the antibody to PPARγ was purchased from Cell Signalling Technology ].

### Cell cycle analysis by propidium iodide staining and flow cytometry

Cells were trypsinized, washed with PBS and fixed with 2% (wt/vol) paraformaldehyde in PBS. They were treated with 0.5 mg/ml RNase A for 1 h at room temperature and incubated with 0.1 mg/ml propidium iodide (Sigma) for 45 min at 37°C. DNA content was determined by flow cytometry (Bio-Rad).

### BrdU labelling and immunofluorescence microscopy

BrdU labeling of hBMSCs and 3T3-L1 cells (kindly provided by Dr. M Daniel Lane, Johns Hopkins University School of Medicine, Baltimore) was performed following the procedure published by Tang [8] with modifications. Cells were plated on to cover-slips and maintained in DMEM containing 10% FBS for several days after confluence and induced to differentiate. Regarding the growth kinetics differennce (hBMSCs have a longer G0/G1 phase than 3T3-L1, the entry of hBMSCs into S phase is ~20h at passage 3 [40]), BrdU for 3T3-L1, BrdU (10 μg/ml) was added at 18 h after induction (during S phase[8]) until 48 h and then shifted to maintain medium (with insulin only); for hBMSCs, BrdU was added at 24 h until 72 h. After differentiation, the cover-slips were fixed in 70% ethanol for 30 min followed by 100% methanol for 10 min at room temperature. The fixed cells were treated for 30 min with 1.5 M HCl, blocked with 0.5% Tween 20 in PBS with 10% FBS for 5 min, incubated with anti-BrdU (1:100, Sigma) or anti-perilipin (1:50, Santa Cruz) primary antibodies in the same buffer overnight, and incubated with FITC/TRITC-conjugated secondary antibodies for 1-2 h. Nuclei were counterstained with 4˜,6-diamidino-2-phenylindole (DAPI). Images were taken on a confocal microscope.

### Adenoviral expression vectors and infection

The adenoviral expression vectors pAd/CMV/V5-DEST (Invitrogen) encoding human C/EBPβ, C/EBPα, PPARγ and Lac Z (control) were constructed according to the manufacturer's protocols. shRNAs for C/EBPα, PPARγ and Lac Z were cloned into pBlock-it (Invitrogen). The sequences of the shRNAs were as follows: C/EBPα, CACCAGGAGGATGAAGCCAAGCAGCTCGAAAGCTGCTTGGCTTCATCCTCCT. PPARγ, CACCGGGTGAAACTCTGGGAGATTCCGAAGAATCTCCCAGAGTTTCACCC. Confluent hBMSCs were infected with the adenovirus at MOI (multiplicity of infection) of 10 for 4 h; the expression of human C/EBPβ, C/EBPα, PPARγ was assessed by real-time PCR at 24 h or by immunoblotting with antibodies against human C/EBPβ, C/EBPα, PPARγ and FLAG at 48 h. For adipocyte differentiation, various combinations of inducers were added to the infected cells for three days. Oil red O staining was used to demonstrate fat lipid accumulation on day eight and western blotting was used to demonstrate FABP4 (422/aP2 in mouse) expression on day four.

### RNAi of C/EBPβ with siRNA

SiRNA oligonucleotides specific for C/EBPβ mRNA (5'-CCCUGCGGAACUUGUUCAAGCAGCU-3') were synthesized by Invitrogen. The silencing effect was verified by real-time PCR for C/EBPβ expression. HBMSCs in 60 mm dishes at 60-70% confluence were transfected with Negative and C/EBPβ siRNA oligonucleotides by using Lipofectamine RNAiMAX (Invitrogen). After 24 h the cells were trypsynized and plated into 35 mm dishes in order to reach confluence immediately. After a further 24 h they were induced to differentiate by three cycles of treatment, and subjected to oil red O staining at day 14.

### Real-time quantitative PCR

Real-time quantitative PCRs were performed with 2× PCR Master Mix (Power SYBR^® ^Green, ABI) on a Bio-Rad Q5 instrument (Bio-Rad). The threshold cycles (Ct) for the target genes and the 18S rRNA control signals were determined in triplicate experiments, and the relative RNA quantity was calculated using the comparative Ct method. Primers were as follows:

18S rRNA: Forward 5'-CGGCTACCACATCCAAGGAA-3', Reverse 5'-GCTGGAATTACCGCGGCT-3'.

C/EBPβ: Forward 5'-GCAAGAGCCGCGACAAG-3', Reverse 5'-GGCTCGGGCAGCTGCTT-3'.

C/EBPα: Forward 5'-AAGAAGTCGGTGGACAAGAACAG-3', Reverse 5'-TGCGCACCGCGATGT-3'.

PPARγ: Forward 5'-GATACACTGTCTGCAAACATATCACAA-3', Reverse 5'-CCACGGAGCTGATCCCAA-3'.

FABP4: Forward 5'-GCTTTGCCACCAGGAAAGTG-3', Reverse 5'-ATGGACGCATTCCACCACCA-3'.

GLUT4: Forward 5'-GCCGGACGTTTGACCAGAT-3', Reverse 5'-TGGGTTTCACCTCCTGCTCTA-3'.

### Statistics

Data were expressed as the mean ± SD of three separate experiments performed in duplicate. Student's *t*-test was used for comparison of results in Figure 4C, Figure 5C and Figure 6C &6H.

## Authors' contributions

SWQ designed the study, carried out the molecular genetics and cell biological studies, performed the statistical analysis, and drafted the manuscript. XL participated in the design of the study and the sequence alignment. YYZ participated in the construction of the vectors. HYH participated in the statistical analysis. YL participated in the cell biological studies. XS performed the confocal scan. QQT conceived the study and participated in its design and coordination, and helped to draft the manuscript. All authors read and approved the final manuscript.

## References

[B1] Hirsch J Batchelor B Adipose tissue cellularity in human obesity Clin Endocrinol Metab 1976 5 299 311 10.1016/S0300-595X(76)80023-0 1085232

[B2] Shepherd PR Gnudi L Tozzo E Yang H Leach F Kahn BB Adipose cell hyperplasia and enhanced glucose disposal in transgenic mice overexpressing GLUT4 selectively in adipose tissue J Biol Chem 1993 268 22243 22246 8226728

[B3] Yu ZK Wright JT Hausman GJ Preadipocyte recruitment in stromal vascular cultures after depletion of committed preadipocytes by immunocytotoxicity Obesity Res 1997 5 9 15 10.1002/j.1550-8528.1997.tb00277.x 9061710

[B4] Crisan M Yap S Casteilla L Chen CW Corselli M Park TS Andriolo G Sun B Zheng B Zhang L Norotte C Teng PN Traas J Schugar R Deasy BM Badylak S Buhring HJ Giacobino JP Lazzari L Huard J Pe'ault B A Perivascular Origin for Mesenchymal Stem Cells in Multiple Human Organs Cell Stem Cell 2008 3 301 313 10.1016/j.stem.2008.07.003 18786417

[B5] Crossno JT Jr Majka SM Grazia T Gill RG Klemm DJ Rosiglitazone promotes development of a novel adipocyte population from bone marrow-derived circulating progenitor cells J Clin Invest 2006 116 3220 8 10.1172/JCI28510 17143331 PMC1679707

[B6] Green H Meuth M An established pre-adipose cell line and its differentiation in culture Cell 1974 3 127 33 10.1016/0092-8674(74)90116-0 4426090

[B7] Green H Kehinde O Spontaneous heritable changes leading to increased adipose conversion in 3T3 cells Cell 1976 7 105 13 10.1016/0092-8674(76)90260-9 949738

[B8] Tang QQ Otto TC Lane MD Mitotic clonal expansion: a synchronous process required for adipogenesis Proc Natl Acad Sci USA 2003 100 44 49 10.1073/pnas.0137044100 12502791 PMC140878

[B9] MacDougald OA Lane MD Transcriptional regulation of gene expression during adipocyte differentiation Annu Rev Biochem 1995 64 345 373 10.1146/annurev.bi.64.070195.002021 7574486

[B10] Tang QQ Grønborg M Huang H Kim JW Otto TC Pandey A Lane MD Sequential phosphorylation of CCAAT enhancer-binding protein beta by MAPK and glycogen synthase kinase 3beta is required for adipogenesis Proc Natl Acad Sci USA 2005 102 9766 71 10.1073/pnas.0503891102 15985551 PMC1175002

[B11] Li X Kim JW Grønborg M Urlaub H Lane MD Tang QQ Role of cdk2 in the sequential phosphorylation/activation of C/EBPbeta during adipocyte differentiation Proc Natl Acad Sci USA 2007 104 11597 11602 10.1073/pnas.0703771104 17601773 PMC1913868

[B12] Darlington GJ Ross SE MacDougald OA The role of C/EBP genes in adipocyte differentiation J Biol Chem 1998 273 30057 60 10.1074/jbc.273.46.30057 9804754

[B13] Freytag SO Paielli DL Gilbert JD Ectopic expression of the CCAAT/enhancer binding protein alpha promotes the adipogenic program in a variety of mouse fibroblastic cells Genes Dev 1994 8 1654 1663 10.1101/gad.8.14.1654 7958846

[B14] Tontonoz P Hu E Spiegelman BM Stimulation of adipogenesis in fibroblasts by PPARγ2, a lipid-activated transcription factor Cell 1994 79 1147 1156 10.1016/0092-8674(94)90006-X 8001151

[B15] Wu Z Rosen E Brun R Hauser S Adelmant G Troy A McKeon C Darlington G Spiegelman B Cross-regulation of C/EBPα and PPARγ controls the transcriptional pathway of adipogenesis and insulin sensitivity Mol Cell 1999 3 151 158 10.1016/S1097-2765(00)80306-8 10078198

[B16] El-Jack AK Hamm JK Pilch PF Farmer SR Reconstitution of insulin-sensitive glucose transport in fibroblasts requires expression of both PPARγ and C/EBPα J Biol Chem 1999 274 7946 7951 10.1074/jbc.274.12.7946 10075691

[B17] Campagnoli C Roberts I Kumar S Bennett P Bellantuono I Fisk N Identification of mesenchymal stem/progenitor cells in human first-trimester fetal blood, liver, and bone marrow Blood 2001 98 2396 2402 10.1182/blood.V98.8.2396 11588036

[B18] In't Anker PS Scherjon SA Kleijburg-van der Keur C Noort WA Claas FH Willemze R Fibbe WE Kanhai HH Amniotic fluid as a novel source of mesenchymal stem cells for therapeutic transplantation Blood 2003 102 1548 1549 10.1182/blood-2003-04-1291 12900350

[B19] Erices A Conget P Minguell JJ Mesenchymal progenitor cells in human umbilical cord blood Br J Haematol 2000 109 235 242 10.1046/j.1365-2141.2000.01986.x 10848804

[B20] De Bari C Dell'Accio F Tylzanowski P Luyten FP Multipotent mesenchymal stem cells from adult human synovial membrane Arthritis Rheum 2001 44 1928 1942 10.1002/1529-0131(200108)44:8<1928::AID-ART331>3.0.CO;2-P 11508446

[B21] Kuznetsov SA Mankani MH Gronthos S Satomura K Bianco P Robey PG Circulating skeletal stem cells J Cell Biol 2001 153 1133 1140 10.1083/jcb.153.5.1133 11381097 PMC2174322

[B22] Tondreau T Meuleman N Delforge A Dejeneffe M Leroy R Massy M Mortier C Bron D Lagneaux L Mesenchymal stem cells derived from CD133-positive cells in mobilized peripheral blood and cord blood: proliferation, Oct4 expression, and plasticity Stem Cells 2005 23 1105 1112 10.1634/stemcells.2004-0330 15955825

[B23] Dominici M Le Blanc K Mueller I Slaper-Cortenbach I Marini F Krause D Deans R Keating A Prockop Dj Horwitz E Minimal criteria for defining multipotent mesenchymal stromal cells. The International Society for Cellular Therapy position statement Cytotherapy 2006 8 315 7 10.1080/14653240600855905 16923606

[B24] Bruder SP Jaiswal N Haynesworth SE Growth kinetics, self-renewal, and the osteogenic potential of purified human mesenchymal stem cells during extensive subcultivation and following cryopreservation J Cell Biochem 1997 64 278 94 10.1002/(SICI)1097-4644(199702)64:2<278::AID-JCB11>3.0.CO;2-F 9027588

[B25] Lehmann JM Lenhard JM Oliver BB Ringold GM Kliewer SA Peroxisome proliferator-activated receptors alpha and gamma are activated by indomethacin and other non-steroidal anti-inflammatory drugs J Biol Chem 1997 272 3406 10 10.1074/jbc.272.6.3406 9013583

[B26] Pittenger MF Mackay AM Beck SC Jaiswal RK Douglas R Mosca JD Moorman MA Simonetti DW Craig S Marshak DR Multilineage potential of adult human mesenchymal stem cells Science 1999 284 143 7 10.1126/science.284.5411.143 10102814

[B27] Pairault J Green H A study of the adipose conversion of suspended 3T3 cells by using glycerophosphate dehydrogenase as differentiation marker Proc Natl Acad Sci USA 1979 76 5138 5142 10.1073/pnas.76.10.5138 291926 PMC413095

[B28] Huang HY Song TJ Li X Hu LL He Q Liu M Lane MD Tang QQ BMP signaling pathway is required for commitment of C3H10T1/2 pluripotent stem cells to the adipocyte lineage Proc Natl Acad Sci USA 2009 106 12670 12675 10.1073/pnas.0906266106 19620713 PMC2722335

[B29] Tang QQ Otto TC Lane MD CCAAT/enhancer-binding protein beta is required for mitotic clonal expansion during adipogenesis Proc Natl Acad Sci USA 2003 100 850 5 10.1073/pnas.0337434100 12525691 PMC298690

[B30] Entenmann G Hauner H Relationship between replication and differentiation in cultured human adipocyte precursor cells Am J Physiol 1996 270 4 Pt 1 C1011 6 8928727 10.1152/ajpcell.1996.270.4.C1011

[B31] Zuk PA Zhu M Ashjian P De Ugarte DA Huang JI Mizuno H Alfonso ZC Fraser JK Benhaim P Hedrick MH Human Adipose Tissue is a Source of Multipotent Stem Cells Mol Biol Cell 2002 13 4279 4295 10.1091/mbc.E02-02-0105 12475952 PMC138633

[B32] Rodriguez AM Elabd C Delteil F Astier J Vernochet C Saint-Marc P Guesnet J Guezennec A Amri EZ Dani C Ailhaud G Adipocyte differentiation of multipotent cells established from human adipose tissue Biochem Biophys Res Commun 2004 315 255 63 10.1016/j.bbrc.2004.01.053 14766202

[B33] Vasseur-Cognet M Lane MD Trans-acting factors involved in adipogenic differentiation Curr Opin Genet Dev 1993 3 238 45 10.1016/0959-437X(93)90029-O 8504249

[B34] Tang QQ Lane MD Activation and centromeric localization of CCAAT/ehnancer binding proteins during the mitotic clonal expansion of adipocyte differentiation Genes Dev 1999 13 2231 2241 10.1101/gad.13.17.2231 10485846 PMC316997

[B35] Wu Z Xie Y Bucher NL Farmer SR Conditional ectopic expression of C/EBP beta in NIH-3T3 cells induces PPAR gamma and stimulates adipogenesis Genes Dev 1995 9 2350 63 10.1101/gad.9.19.2350 7557387

[B36] Hamm JK Park BH Farmer SR A role for C/EBPbeta in regulating peroxisome proliferator-activated receptor gamma activity during adipogenesis in 3T3-L1 preadipocytes J Biol Chem 2001 276 4 71 1846 10.1074/jbc.M100797200 11279134

[B37] Lin FT Lane MD CCAAT/enhancer binding protein alpha is sufficient to initiate the 3T3-L1 adipocyte differentiation program Proc Natl Acad Sci USA 1994 91 8757 61 10.1073/pnas.91.19.8757 8090719 PMC44685

[B38] Kim JB Wright HM Wright M Spiegelman BM ADD1/SREBP1 activates PPARgamma through the production of endogenous ligand Proc Natl Acad Sci USA 1998 95 4333 7 10.1073/pnas.95.8.4333 9539737 PMC22489

[B39] Sandouk T Reda D Hofmann C Antidiabetic agent pioglitazone enhances adipocyte differentiation of 3T3-F442A cells Am J Physiol 1993 264 6 Pt 1 C1600 8 8333508 10.1152/ajpcell.1993.264.6.C1600

[B40] Zhang Y Li CD Jiang XX Comparison of mesenchymal stem cells from human placenta and bone marrow Chinese Medical Journal 2004 117 882 887 15198892

